# Supplementary material for: Prevalence of Germline Mutations in Cancer Predisposition Genes in Patients with Pancreatic Cancer or Suspected Related Hereditary Syndromes: Historical Prospective Analysis
Source: Cancers (Basel). 2023 Mar 20;15(6):1852. doi: 10.3390/cancers15061852 (PMC10047356; doi:10.3390/cancers15061852)
Supplement: Supplementary file 1 [file cancers-15-01852-s001.zip › cancers-2214861-supplementary.pdf]

Table S1. Genes included in the germ-line testing of the study.

Genes included in the germ-line testing
APC
ATM
BRCA1
BRCA 2
BAP1
CDK4
CDKN2A
CHEK2
EPCAM
MITF
MLH1
MSH2
MSH6
PALB2
PMS2
SMAD4
STK11
FANCM
TP53
